# Feeding, stooling and sleeping patterns in infants with colic - a randomized controlled trial of minimal acupuncture

**DOI:** 10.1186/1472-6882-11-93

**Published:** 2011-10-11

**Authors:** Kajsa Landgren, Nina Kvorning, Inger Hallström

**Affiliations:** 1 Department of Health Science, Faculty of Medicine, Lund University, P.O. Box 157, SE-221 00 Lund, Sweden; 2 Department of Anesthesiology and Intensive Care, Hospital of Southwestern Jutland, Finsensgade 35, DK-6700 Esbjerg, Denmark

## Abstract

**Background:**

The aim was to describe the feeding- and stooling patterns of infants with colic and evaluate the influence of minimal acupuncture.

**Methods:**

A prospective, randomized, controlled, blind clinical study was conducted at a private acupuncture clinic in Sweden. 90 otherwise healthy 2-8 weeks old infants, born after gestational week 36, fulfilling the criteria for infantile colic and not medicated with dicyclomine, were included. 81 infants went through a structured program consisting of six visits to the clinic, twice weekly. Infants randomized to receive acupuncture were given minimal, standardized acupuncture for two seconds in LI4. Frequency and size of stooling, as well as duration of, and intervals between, feeding sessions were reported by parents in a diary. Parental assessment of sleep and comments on stooling and side effects were collected in a questionnaire.

**Results:**

At baseline when the mean age was five weeks, infants in both groups were fed a median of eight times/day, 148 min/day, with considerable variations. No differences were found between groups in the frequency and duration of feeding during the intervention weeks. Furthermore there were no significant differences between the groups regarding the frequency of stooling, neither at baseline, at which point the infants of both groups had bowel movements 4.2 times/day, nor during the intervention weeks. There was an expected decrease in frequency of stooling in both groups, reaching 2.1 (p = 0,001) in the acupuncture group and 3.1 (p < 0,001) in the control group. The groups differed regarding large bowel movements which decreased linearly in the control group (p = 0,011) but not in the acupuncture group (p = 0,787). More parents in the acupuncture group than in the control group (28% and 15% respectively, p = 0.006) experienced the infant's sleep to be "better" or "much better." No other significant differences were found. However, parents described a normalized stooling and experienced an improvement in colic in their infants more frequently in the acupuncture group than in the control group.

**Conclusions:**

Infants with colic in the present study had a higher frequency of stooling than reported internationally in healthy infants. Minimal acupuncture had no major effect on feeding, stooling and sleep, although a minor effect of minimal acupuncture on stooling and sleep cannot be ruled out.

**Trial registration:**

ClinicalTrials.govID NCT00860301

## Introduction

Infantile colic, with a prevalence of approximately 10% of infants[1], is often related to gastrointestinal factors by the parents[2] although the aetiology is unclear[3]. Few studies describe feeding frequency and bowel habits during the first months in healthy infants[4-10], even fewer describe this in infants with colic[11,12]. Possible effect of acupuncture on feeding, stooling and sleeping patterns in infants suffering from colic has not been investigated previously.

## Background

Parents are encouraged to feed their newborn baby when and for as long as the baby desires in order to adjust the natural control of appetite, maternal milk production or amount of formula. 6-8 meals/day is the postulated standard [4]. We have not been able to find any scientific data as to how often infants are actually fed. Age is the factor that influences stooling frequency most. Healthy infants have bowel movements approximately four times/day during their first 1-2 weeks [5-7]. At the age of one month infants are registered to have 2.2 stoolings/day [7], approximately three stoolings/day[6,8-10] and in one study six stoolings/day [11]. Following the first weeks there is a radical decrease in stooling frequency till the age of two months when stooling frequency was reported to be one per day [11], 1.8 [7,10] and 2.2 [9] times/day. At three months the mean frequency had decreased to one per day [11], 1.25/day[13] and approximately 1.7/day [7,10]. Breast feeding has been reported to increase the frequency of stooling [11,14,15]. Two trials measured the difference between the bowel movements of colicky and non-colicky infants: one reported that infants with symptoms of colic during the first two months had less frequent bowel movements [11], the other that there was no difference[12].

Parents of infants with colic correlated crying to stomach aches and a disturbed gut function [2,16]. In a qualitative study they remarked that their infants had bowel movements more than ten times/day or hardly at all and that the stools were green, explosive and foul-smelling [2].

Colicky symptoms have been linked to feeding problems and disturbed sleep [17]. In two randomized controlled studies minimal acupuncture in LI4 in infants with colic resulted in a reduction of fussing and crying [18,19]. Possible explanations could be a reduction of pain as shown in adults [20], a beneficial effect on other visceral symptoms such as nausea which has been reduced by acupuncture in adults [21,22] and in children [23], an altered gastric motility [24] or changed gastric emptying as shown in adult patients with motility disorders [25]. Furthermore acupuncture affected constipation in children [26] even though gastric motility in healthy adult humans was not altered [27]. Finally a sedative effect of acupuncture could explain the reduction of colic as it has been demonstrated to promote sleep in adults [28].

The aim of this study was to describe the feeding and stooling patterns of infants with colic. A second aim was to evaluate the influence of minimal acupuncture.

## Methods

### Design

The study was performed at a private acupuncture clinic in Sweden comparing acupuncture with no acupuncture in a prospective, clinical, blinded, randomized, controlled trial (RCT). The primary outcome of the RCT was the frequency of fussing and crying of the infants, described in a separate article [19]. The secondary outcomes were the feeding, stooling and sleeping patterns of the infants as presented in the present article. The study was approved by the Regional Ethical Review Board (Dnr 583/2005).

### Participants

**Figure 1:**
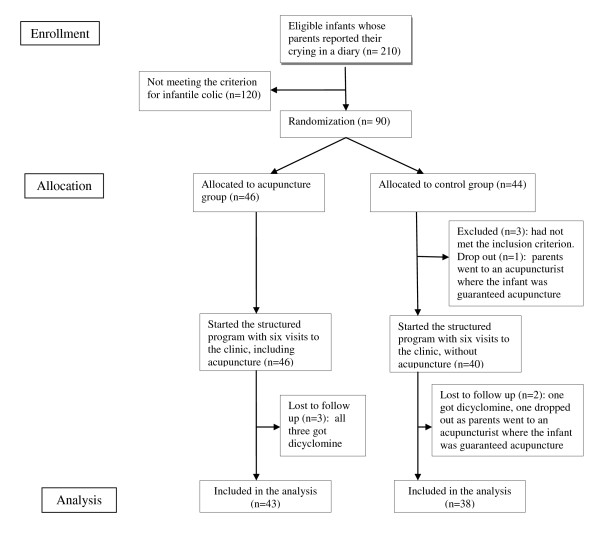
**Flow chart of infants through trial**.

Parents of otherwise healthy 2-8 week old infants, born after gestational week 36, never medicated with dicyclomine and searching help for excessive crying were invited to participate. After giving written informed consent the parents of 210 infants reported the crying and fussing of their infants in a diary for at least three days to assess whether or not the infants fulfilled the modified Wessel-criteria for colic: "crying/fussing for at least three hours a day, occurring three days or more in the same week" [1]. In the diary the parents also reported the feeding and stooling habits of their infants. During the registration period exclusion of cow's milk from the infant's diet was recommended if this had not been tried previously. Of these 210 infants 120 were not included as they cried less than the stipulated hours. Some of them may have improved as they were no longer exposed to cow's milk protein. The 90 infants fulfilling the criteria for crying were randomized and 81 completed all three intervention weeks (Figure 1).

### Randomisation and intervention

Infants went through a structured program consisting of six visits to an acupuncture clinic, twice weekly. (Two infants in the acupuncture group went to the clinic only five times as the parents considered the colic to be cured. They were included as the parents continued to register in the diary.) At each visit the parents met a nurse who asked standardized questions and gave standardized support and advice. At every visit the infant was carried to a treatment room and left alone for five minutes with a certified acupuncturist who was also a registered nurse and well qualified in the care of children. She administered minimal, standardized acupuncture to the infants allocated to receive acupuncture. This was performed unilaterally (left and right hands were used alternately for each child), for two seconds at an approximate depth of two millimeters in the point LI4 of the first dorsal interosseal muscle of the hand, innervated by the ulnar nerve by sensory and autonomic, mainly sympathetic, fibres. An acupuncture needle, 0.20 × 13 mm, was used (Vinco Micro Clean). Infants in the control group were treated similarly except for the needle insertion.

### Blinding

The nurse who met the parents was blinded to the randomization. The acupuncturist performed the randomization and never met the parents. Parents were informed that acupuncture does not necessarily cause the infant to cry and that the very thin needle usually would not cause bleeding or a visible mark on the skin.

### Outcome and assessments

The parents made notes every 5-minute when applicable, for 24-hours of their infants' crying, feeding and stooling in a diary modified from Barr et al [29] and Canivet [30]. Reports were made for at least three days during the baseline week and daily during the three intervention weeks. Parents marked large bowel movements with "B" and small bowel movements, "a stain of feces in the nappy," with "b." Parents also reported when, and for how long, the infant was eating. At inclusion parents were asked if the appetite of the infant was "bad," "good" or "gluttonous." From the second to the sixth visit parents completed a questionnaire modified from Reinthal et al [18] in which they were asked "Has the stooling of your infant changed, yes or no?" Parents could even describe the situation in their own words, responding to the questions "If so, how?" and "Have you detected any symptom that you think can be related to the acupuncture treatment? If so what?" In the same questionnaire parents could describe the present sleep of their infant in a five-point scale as much worse, worse, as before, better or much better. At the last visit parents answered the question "Do you think your baby's colic is much worse, worse, the same, a little bit better or much better than three weeks ago?"

### Statistical and qualitative analyses

The power calculation was based on assumptions of how acupuncture would affect the primary outcome of crying. If 50% of the infants would go into spontaneous remission without treatment and 75% with acupuncture, 40 patients per group were needed in order to have 90% chance of detecting a significant difference in remission rate at a two-sided 5% level. The statistical software SPSS™ version 17 (SPSS Inc., Chicago, IL) was used for calculations. As the Kolmogorov-Smirnov test showed non-normal distribution for core variables, the Mann-Whitney U test was used to analyze differences between groups at each time point. Changes within the groups over baseline and the three intervention weeks were analyzed with Friedman test. P-values < 0.05 were considered statistically significant.

Answers concerning changed stooling and possible side effects were summarised and analyzed with content analysis at a manifest level meaning that the visible content in the text was coded [31]. Similar remarks were grouped together into one code, codes with similar content were grouped into categories and the amount of remarks was registered. The coding was done by the first author and subsequently checked by the third author to ensure intercoder reliability [31].

## Results and discussion

There were no significant differences between groups, neither regarding the background characteristics of the infants (Table 1), nor in feeding and stooling patterns, during the baseline registration week (Table 2).

**Table 1:** Baseline demographic data

Background characteristics	Infants starting the intervention (N = 86)		Infants completing 3 weeks (N = 81)	
	**Acupuncture group (n = 46)**	**Control group (n = 40)**	**Acupuncture group (n = 43)**	**Control group (n = 38)**

Firstborn, n (%)	22 (48)	22 (55)	21 (48)	21 (55)

Gender, female, n (%)	22 (48)	19 (48)	21 (50)	19 (50)

Gestational age, weeks. Mean (SD)	39.2 (1.5)	39.5 (1.3)	39.3 (1.4)	39.5 (1.3)

Age when colic started, weeks. Mean (SD)	1.9 (1.3)	1.5 (1.0)	2 (1.3)	1.5 (1)

Age at inclusion, weeks. Mean (SD)	5.0 (1.9)	5.3 (1.7)	5.1 (1.9)	5.2 (1.6)

Solely breastfed, n (%)	35 (76)	26 (65)	32 (74)	25 (66)

Having a parent and/or sibling with food intolerance/allergy, n (%)	17 (37)	18 (45)	15 (35)	17 (45)

Having a parent and/or sibling who had had infantile colic, n (%)	29 (63)	23 (58)	25 (58)	20 (53)

Have tried simeticone, n (%)	43 (93)	37 (92)	40 (93)	35 (92)
With no effect, n (%)	28 (61)	25 (63)	26 (60)	23 (61)
With uncertain effect, n (%)	15 (33)	12 (30)	14 (33)	12 (32)

Have tried lactobacillus reuteri, Semper Magdroppar ^®^, n (%)	6 (13)	7 (18)	6 (14)	6 (16)
With no effect, n (%)	4 (9)	7 (18)	4 (9)	6 (16)
With uncertain effect, n (%)	2 (4)	0 (0)	2 (5)	0 (0)

Have tried a diet free from cow'rotein without effect, n (%)	33 (72)	32 (80)	31 (72)	30 (79)
Changed to this diet during the intervention weeks, n (%)	1 (2)	1 (3)	1 (2)	1 (3)
Not ensured if this diet is tried, n (%)	12 (26)	7 (18)	11 (26)	7 (18)

Received antibiotics, either through mother's medication or by own intake, n (%)	14 (30)	10 (25)	12 (28)	9 (24)

**Table 2:** Stooling and feeding during the baseline week and the three intervention weeks (Mann-Whitney U test was used.)

	Baseline		First intervention week			Second intervention week			Third intervention week		
**Categories of feeding or stooling**	**Acupuncture group** **(n =43)**	**Control group** **(n =38)**	**Acupuncture group** **(n =43)**	**Control group** **(n =38)**	**P value**	**Acupuncture group** **(n =43)**	**Control group** **(n =38)**	**P value**	**Acupuncture group** **(n =43)**	**Control group** **(n =38)**	**P value**

Feeding, times/day Median (quartiles, 25 and 75%)	8,0 (7.2-10.0)	8,0 (6.9-9.1)	8,1 (7.0-9.7)	7,9 (6.9-9.6)	0.602	8,1 (7.0-9.7)	7,8 (6.7-8.9)	0.435	8,4 (6.9-9.6)	7,7 (6.5-9.6)	0.660

Feeding, minutes/day Median (quartiles, 25 and 75%)	155 (113-193)	140 (118-178)	154 (107-196)	157 (114-189)	0.723	153 (112-186)	164 (114-177)	0.748	140 (108-196)	145 (104-188)	0.854

Stoolings, total times/day Median (quartiles, 25 and 75%)	4,1 (2.2-6.0)	4,3 (2.7-6.5)	3,9 (2.0-5.4)	4,1 (1.9-5.9)	0.698	2,6 (1.3-4.9)	3,6 (1.6-5.2)	0.463	2,1 (1.1-4.7)	3,1 (1.0-4.6)	0.902

Stoolings, large/day Median (quartiles, 25 and 75%)	2,5 (1.3-3.7)	2,5 (1.3-4.3)	2,3 (1.3-3.4)	2,6 (1.4-3.9)	0.538	1,7 (1.0-3.4)	2,2 (1.1-3.9)	0.570	1,7 (1.1-3.4)	1,8 (0.9-3.6)	0.846

Stoolings, small/day Median (quartiles, 25 and 75%)	1,2 (0.3-3.0)	1,8 (0.4-2.7)	1,0 (0.4-2.0)	1,1 (0.3-2.0)	0.827	0,7 (0.1-1.3)	0,7 (0.1-1.7)	0.546	0,3 (0.0-1.7)	0,6 (0.0-1.6)	0.583

### Feeding

During baseline and the three intervention weeks infants in both groups were fed approximately eight times/day with a variation between 5.3 and 14.2 times/day (Table 2), placing this group in the upper level of the previously reported norm of 6-8 times/day [4]. At inclusion the appetite of the infant was described as "gluttonous" by 56% of the parents, as "good" by 42% and as "bad" by 2% with no difference between the groups. This correlates well to the thesis that infants with colic may be comforted with food and thus be fed more often than healthy infants [2]. Furthermore infants who are breastfed, and fed with short intervals consume less of the high-fat hindmilk and more of the low-fat milk which has a more rapid stomach transit time which, in turn, may result in short intervals between meals [4] and increase feeding problems [17].

The duration of feeding in the present study was approximately 148 minutes/day, with considerable variations (min 49, max 458). There were no statistical differences between groups in frequency or duration of meals.

### Stooling

At baseline when the infants in the present study had a mean age of five weeks they had bowel movements 4.1 times/day (acupuncture group) and 4.3 times/day (control group). These frequencies are higher than the 2.2 - 3 times/day reported in the majority of previous prospective studies in healthy infants at the age of one month [6-10] and contra dictionary to the findings of Tunc *et al*. who reported less frequent stooling in colicky infants compared to non-colicky infants [11]. However, as different feeding habits and living conditions may affect stooling, a comparison between trials performed in different countries should be validated in local studies. Many different influences are possible. For example, the frequency of breastfeeding and the consumption of antibiotics which may increase stooling frequency [4] could well vary in the countries in which data have been collected (Sweden, Australia, Germany, Thailand, Turkey, UK and the USA).

Frequency of stooling varied widely between infants in the present study, from the mean of 0,1 to the mean of 12,4 times/day (Table 2) as it has been observed in other studies [5-11]. There were no significant differences between groups in the mean frequency of stooling (Table 2) and there was a decline in both groups during the intervention weeks (p = 0,001 in the acupuncture group and p < 0,001 in the control group). This decrease was expected as previous prospective studies report a decrease in stooling frequency between the first month (2.2 - 6 stoolings/day) and the second month (1 - 2.2 stoolings/day) [7,9-11]. In the present study, the frequency in the third intervention week, mean age eight weeks, was 2.1 times/day in the acupuncture group and 3.1 in the control group. Thus the frequency in the control group remained higher than average whereas infants in the acupuncture group reached a frequency reported in earlier studies of healthy infants. Possibly this could be the result of a normalized gastrointestinal function in the acupuncture group.

The mean value of large bowel movements decreased linearly in the control group (p = 0,011) but not in the acupuncture group (p = 0,787).

### Changed stooling patterns and possible side effects

In the acupuncture group parents commented on changes in stooling habits of their infants 56 times as compared to 25 times in the control group. Comments on changes in the stooling frequency and possible side effects consisted of a total of 271 remarks (168 from the acupuncture group, 103 from the control group). The amount of remarks reported from each group, codes and categories are presented in Table 3. Close to twice as many parents in the control group remarked that the infants' stools were "more watery" (22 to 12). In contrast almost three times as many parents in the acupuncture group (16 to 6) remarked that the infant's stools were "more firm." The word "normalized" or a similar word was mentioned 22 times in the acupuncture group and 8 times in the control group. Parents in the acupuncture group gave 16 comments coded into "Can defecate/break wind easier/without help" compared to none in the control group. Comments on stooling, and the fact that the mean value of large bowel movements decreased linearly in the control group but not in the acupuncture group, could indicate that the minimal acupuncture used in this trial actually affected the bowel movements, supporting reports from other trials on autonomic effects of acupuncture on the gastrointestinal system [24,25].

**Table 3:** Changes in stooling pattern and possible side effects of intervention as described by the parents in a questionnaire from the second till the sixth visit at the clinic

Categories	Codes	Number of remarks in the acupuncture group	Number of remarks in the control group
Changed stooling	Less frequent	30	17

	More frequent	26	8

	More watery	12	22

	More firm	16	6

	Changed colour	22	15

	Changed odour	7	10

	Normalized, "less hassle and conkout with the stomach"	22	8

	Can defecate and/or break wind easier/without assistance	16	0

Other gastro-intestinal changes	Can belch easier	1	0

	Increased salivation	0	1

	More vomiting	3	4

	Rumbling in the stomach	2	4

Other symptoms	More crying/less crying	3/1	3/1

	More restless after the visit	3	0

	Wants to eat more often	1	0

	Increased skin symptoms	2	1

	Slept a lot	1	3

The categories, codes and frequency of remarks on a certain topic in each group are shown in this table. Each remark has only been registered once in each questionnaire

### Sleeping and progression of colic

Sleep was rated as "better" or "much better" (see Table 4) more frequently in the acupuncture group than in the control group. During the second week parents in the acupuncture group reported "better" or "much better" 26 times (constituting 30% of the answers) compared to six times in the control group (8% of the answers). During the study period significantly more parents in the acupuncture group (28% compared to 15% in the control group, p = 0.006) evaluated the infant's sleep as "better" or "much better." More parents in the acupuncture group experienced an improvement in colic during the study time at the last visit (Table 4).

**Table 4:** Changes in sleep and development of colic during the three intervention weeks

	Sleep #		Colic##	
	**Acupuncture group (43)**	**Control group (38)**	**Acupuncture group (43)**	**Control group (38)**

Much better, n (%)	16 (8)	3 (2)	25 (58)	13 (34)

Better, n (%)	43 (20)	24 (13)	17 (40)	17 (45)

As before, n (%)	102 (49)	116 (63)	1 (2)	7 (18)

Worse, n (%)	41 (20)	35 (19)	0	1 (3)

Much worse, n (%)	8 (4)	6 (3)	0	0

Total, n (%)	210 (100)	184 (100)	43 (100)	38 (100)

# comments collected twice weekly during week 1 and 2 and once week 3

## comments collected at the last visit, week 3

No definite statistical conclusions can be made on independent variables like the comments of the parents above. However, the parents perception of normalized stooling, better sleep and improvement of the colic are in line with the results reported earlier: the infants in the acupuncture group cried and fussed less and the mean value for crying was below the limit for colic after the first intervention week [19]. The infants in the acupuncture group reached normal levels for their age of the stooling frequency in the third intervention week (Table 2), ie < 2.2 stoolings/day, [7,9-11]. The largest reduction of both crying and stooling frequency was measured after the first acupuncture treatment. However, the differences in feeding and stooling patterns between the groups are not significant in the majority of the variables and the present study cannot support a simple correlation between reduction of crying and an improved regulation of these. The effect of acupuncture may as well be of a different origin such as spasmolytic or sedative.

### Strengths and limitations

One strength of the present study is that quantitative and qualitative methods were combined to detect even subtle changes that are not so easily captured, such as characteristics of bowel movements other than the two variables of frequency and size[32]. Furthermore, frequency and size of bowel movements were reported meticulously in the diaries kept by all parents during the three week study period. Another strength is that parents of all of the infants had been recommended to try a five-day period of not exposing the infant to cow's milk protein during baseline. Infants improving from this were not included in the study reducing thereby the number of infants with an allergy to cow's milk protein to a minimum.

Limitations are the lack of precise measurement of the stools in milligrams, and the fact that the infant's sleep was described by multiple choise alternatives but not measured in minutes in the diary. Furthermore, parents were not asked to evaluate the infant's sleep during the baseline period. Special feeding habits and living conditions, possibly affecting stooling, were not registered. The parents' experiences are important but subjective and should be interpreted with care.

In this study only one, unilateral, acupuncture point, minimally stimulated, was investigated. As different points and stimulation techniques have been demonstrated to have different effects on gastrointestinal symptoms [33-36] the results of the present study cannot be generalised to a situation in which other points or to stronger stimulation are used.

Power calculation was done on the variable crying, reported in an earlier article [19] and not on variables as stooling or feeding which might have resulted in another number of participating infants. Another limitation is that no correlation analyses was done to see if the individual crying and stooling patterns were correlated in each child, and if the experience of normalized stooling or general improvement according to the parent was correlated to reduced crying in the infant.

## Conclusion

This article reports, for the first time, the feeding and stooling patterns of Swedish infants with colic. Feeding and stooling habits are important topics for many new parents. A description of these patterns can be a valuable tool in everyday clinical practice. Infants with colic in the present study had a higher frequency of stooling than reported internationally in healthy infants.

Minimal acupuncture in the point LI4 twice a week for 3 weeks only showed a minor difference in the frequency of stooling between the groups. The parents in the acupuncture group more frequently commented on a changed and normalized stooling in their infants, and more frequently reported improvement of sleep and colic. As the correlation between relief of colic symptoms and frequency in feeding and stooling is weak there may be other explanations for the effect on crying induced by acupuncture. Further studies are requested to clarify the mechanism of acupuncture in colic.

## Competing interests

The authors declare that they have no competing interests.

## Authors' contributions

KL and IH contributed to planning the study. KL collected data. All three authors contributed to analysing data and writing the article. IH contributed to supervision. All authors read and approved the final manuscript.

## Pre-publication history

The pre-publication history for this paper can be accessed here:

http://www.biomedcentral.com/1472-6882/11/93/prepub
